# A Model Example: Coexisting Superior Mesenteric Artery Syndrome and the Nutcracker Phenomenon

**DOI:** 10.1155/2015/649469

**Published:** 2015-09-03

**Authors:** Rebecca Nunn, Jaimie Henry, Alistair A. P. Slesser, Rukshan Fernando, Nebil Behar

**Affiliations:** ^1^Department of General Surgery, Chelsea and Westminster Hospital, 369 Fulham Road, London SW10 9NH, UK; ^2^Department of Radiology, Chelsea and Westminster Hospital, 369 Fulham Road, London SW10 9NH, UK

## Abstract

Superior mesenteric artery (SMA) syndrome is a rare cause of gastrointestinal obstruction, caused by external compression of the third part of the duodenum by the SMA. It may be associated with the Nutcracker phenomenon: external compression of the left renal vein. To our knowledge, there are few reports in the literature describing the coexistence of these two conditions and so we take this opportunity to highlight a rare cause of the acute abdomen that might otherwise be overlooked in cases of nonspecific abdominal findings and potentially unremarkable initial investigations. We report a case of SMA syndrome and Nutcracker phenomenon in a 19-year-old female who presented to our emergency department with a short history of epigastric pain and emesis. The SMA syndrome is thought to develop as the result of an abnormally narrow angle between the proximal SMA and the aorta, for which a number of predisposing factors have been described. Surgical options exist; however, the SMA syndrome is typically managed conservatively in the first instance, consistent with the approach described in this case. The Nutcracker phenomenon may give rise to the Nutcracker syndrome in the presence of typical clinical manifestations; however, these did not feature in this case.

## 1. Introduction

Superior mesenteric artery (SMA) syndrome (Wilkie's syndrome) is a rare condition whereby external compression of the third part of the duodenum by the SMA results in duodenal obstruction. Nutcracker phenomenon refers to compression of the left renal vein by the SMA, an isolated radiological finding, with potential clinical manifestations such as left flank pain, haematuria, and left-sided varicocele. We describe a rare emergency presentation of SMA syndrome with coexisting Nutcracker phenomenon.

## 2. Case Presentation

A 19-year-old Caucasian female fashion student and model presented as a surgical admission complaining of a twelve-hour history of severe epigastric pain which was associated with emesis and anorexia. On further questioning, the patient described a history of insidious symptoms over three years, characterised by postprandial abdominal pain, bloating, and occasional vomiting, without significant weight loss. There was an absence of any previous medical or psychiatric diagnoses and no previous abdominal surgery. The patient denied any intentional dieting or weight loss over this period. There was no history of fever or foreign travel.

**Figure 1:**
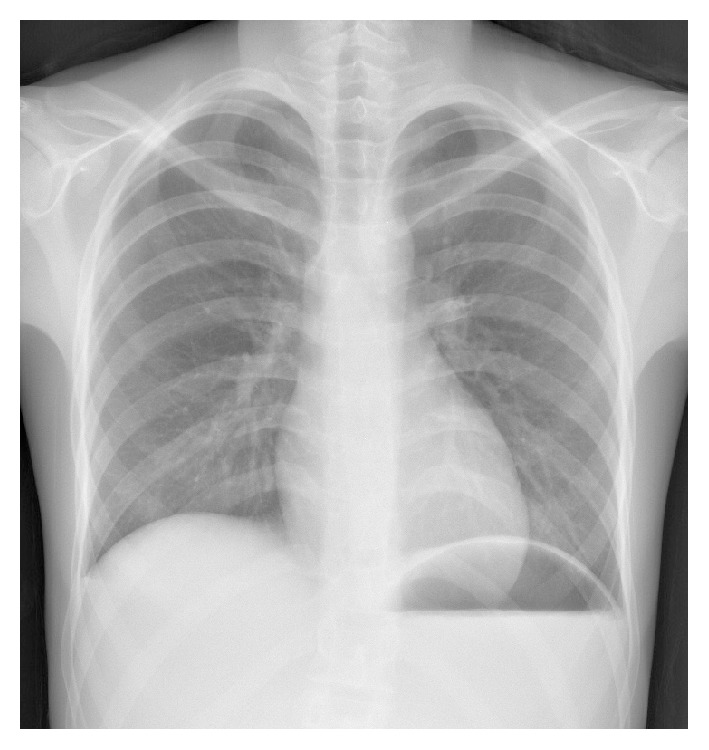
Erect chest radiograph demonstrating gastric air-fluid level.

Clinical examination revealed an underweight and pale patient. The patient was clinically stable and abdominal examination revealed a soft abdomen with epigastric tenderness. Initial blood test results were unremarkable: white cell count, 6.1 × 10^9^/L (4.2–11.2); C-reactive protein, <0.2 mg/L; haemoglobin, 145 g/L (114–150); sodium, 144 mmol/L (133–146); potassium, 3.9 mmol/L (3.5–5.3); eGFR, 74 mL/min (MDRD formula); amylase, 92 IU/L; and lactate, 1.45 mmol/L (0.6–2.5). Urinalysis was negative for blood, leukocytes, and nitrites, with a negative pregnancy test. An erect chest radiograph demonstrated an air-fluid level in the stomach (Figure 1).

**Figure 2:**
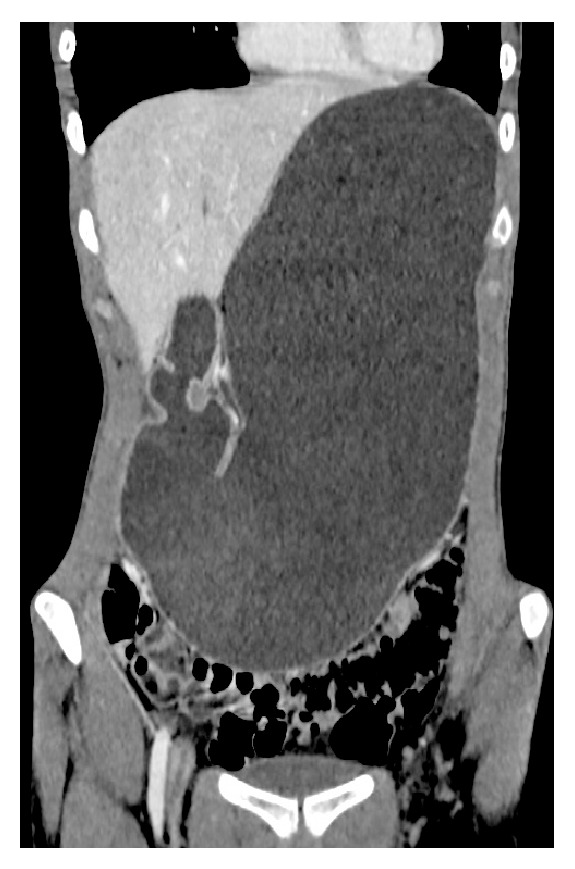
Coronal CT section demonstrating gross distension of the stomach.

**Figure 3 fig3:**
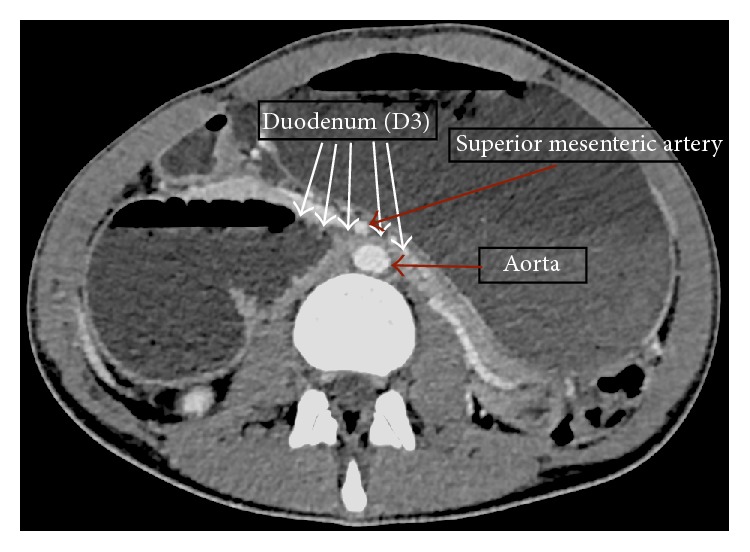
Axial CT section demonstrating the anatomy of SMA syndrome.

**Figure 4:**
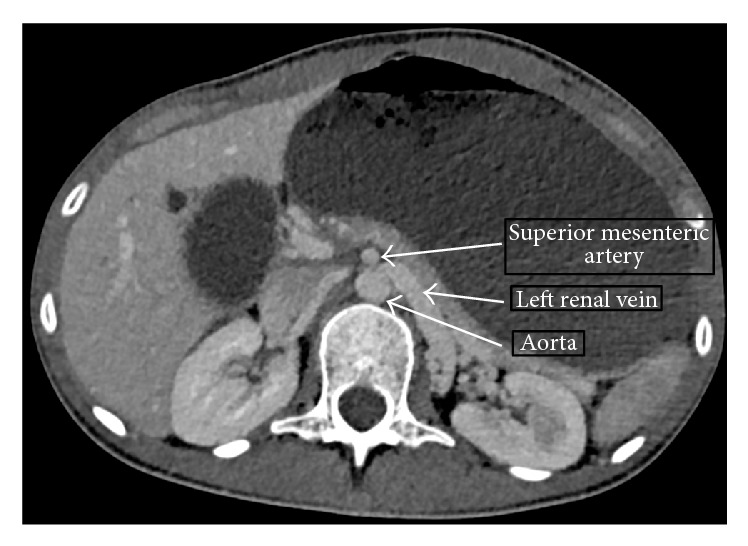
Axial CT section demonstrating the anatomy of Nutcracker phenomenon.

The disparity between the patient's clinical appearance, biochemical results, and initial imaging gave cause for clinical concern, and so the patient underwent a contrast enhanced computerised tomography (CT) scan (Figures 2 [Fig fig3]–4). The CT scan revealed a grossly distended stomach, dilated proximal duodenum, and a narrowing of the third part of the duodenum (D3) between the angle of the superior mesenteric artery and abdominal aorta. There was also narrowing of the left renal vein underlying the superior mesenteric artery.

**Figure 5:**
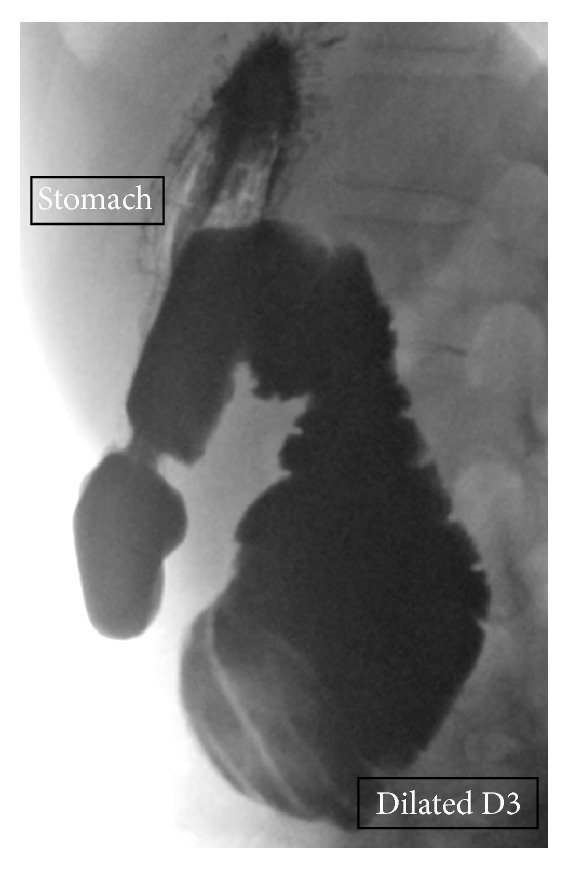
Lateral view of contrast study demonstrating dilated D3.

**Figure 6:**
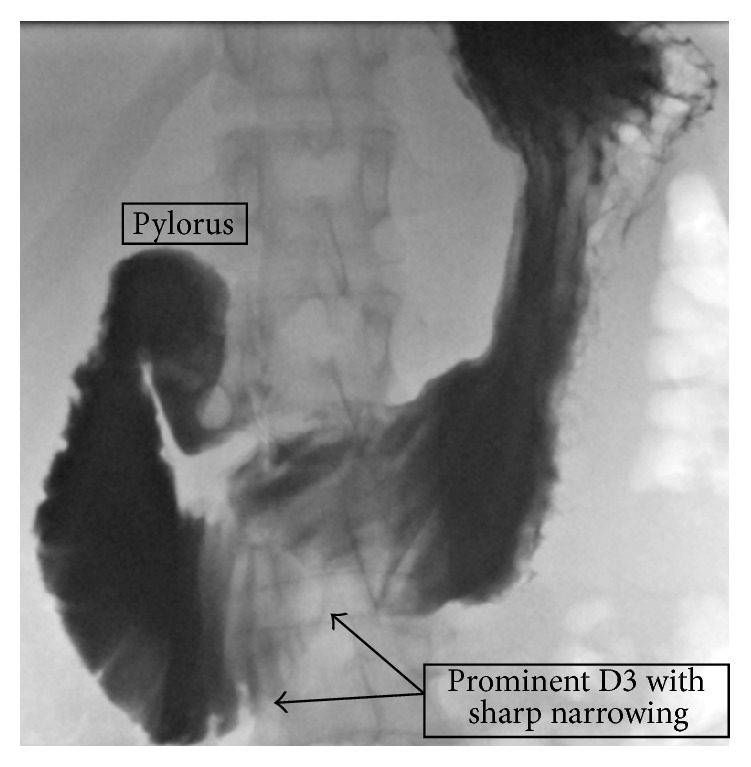
Antero-posterior view of contrast study demonstrating a dilated D3 with sharp narrowing mid segment.

A diagnosis of acute-on-chronic SMA syndrome with coexisting Nutcracker phenomenon was made. Gastric decompression was instituted with a nasogastric tube insertion, which immediately drained 900 mL of bilious fluid. The patient was advised to lie with her knees flexed towards the chest in the left lateral decubitus position and was resuscitated with intravenous fluids. Nutritional support was initially provided through combined TPN (total parenteral nutrition) and enteral means, with a multidisciplinary team approach including input from general surgery, gastroenterology, and dietetics. Potential thromboembolic risk secondary to left renal vein compression was addressed with thromboembolic stockings and prophylactic low weight molecular heparin. After 12 days, the patient was discharged with outpatient followup. Results of further investigations included an oesophageal gastroduodenoscopy (OGD) which showed a normal duodenal cap and second part of the duodenum (up to D3), a normal gastric emptying study, and a barium meal and follow through study with findings consistent with the diagnosis of SMA syndrome. The latter two studies were performed on an outpatient basis (Figures 5 and 6).

## 3. Discussion

In 1842, Rokitansky first described a syndrome of external compression of the third part of the duodenum by the superior mesenteric artery with resultant obstruction, now widely referred to as superior mesenteric artery (SMA) syndrome [1]. Patients may present acutely with signs and symptoms of duodenal obstruction or with a more chronic picture of vague postprandial symptoms such as abdominal pain, nausea, vomiting, and weight loss [1]. Incidence is estimated at 0.013–0.3% of the general population, with higher incidences reported in those post-scoliosis surgery and burns patients [1, 2]. Females are most commonly affected, with the majority of patients aged between 10 and 39 years [1, 3]. Dieting and eating disorders have also been previously described as risk factors [1].

The pathophysiology of SMA syndrome is widely believed to result from an abnormally acute angle between the proximal part of the SMA and the abdominal aorta (reduced to 6–16° compared to the typical 38–56°) as the SMA crosses the third part of the duodenum anteriorly, which then leads to duodenal compression and a reduction in mean aortomesenteric distance (reduced to 2–8 mm compared to a typical mean of 10–28 mm) [1]. Several mechanisms to account for a reduction in the aortomesenteric angle have been proposed [1, 4]:

- Loss of fat and lymph tissue at the origin of the SMA, reducing the ability to “cushion” the third part of the duodenum and protect it from compression, typically associated with rapid weight loss and altered metabolic states.
- Anatomical anomalies such as a short suspensory ligament of Treitz resulting in high suspension of the duodenojejunal flexure, intestinal malrotation, or increased lumbar lordosis.
- Pressure from external fittings such as body casts.
- Postoperative complications (associated operations include total proctocolectomy and ileoanal pouch anastomosis, scoliosis surgery, and abdominal aortic aneurysm repair).

External compression of the left renal vein noted on the scan is indicative of the Nutcracker phenomenon. This commonly occurs as the left renal vein traverses between the abdominal aorta and the superior mesenteric artery (*anterior Nutcracker*), with resulting impaired venous outflow. In a retroaortic left renal vein, compression may occur between the aorta and vertebral body (*posterior Nutcracker*) [5, 6]. The pathophysiological basis for the differing clinical and subclinical phenotypes of Nutcracker phenomenon/syndrome is poorly characterised in the literature [6]. In contrast to the evident duodenal obstruction as a result of the SMA syndrome, the typical clinical manifestations that define Nutcracker syndrome (including left flank pain, haematuria) were absent in this case [5, 6]. Therefore, the terminology Nutcracker phenomenon is used. The coexistence of both SMA and Nutcracker syndromes has been rarely reported in the literature to date [2, 7–9].

Clinical diagnosis of SMA syndrome remains challenging because of both the nonspecific range of symptoms and the rarity of the condition. Diagnosis is made through imaging, although the gold standard modality and diagnostic criteria remain unclear [1, 3]. An OGD is often undertaken to rule out any intraluminal pathology that may be secondary to SMA syndrome (such as peptic ulcer disease secondary to reflux), or mimicking a presentation of SMA syndrome [1]. Nutcracker syndrome is similarly diagnosed through imaging but this is often prompted by the clinical manifestations of the disease.

No randomised clinical trials exist regarding management of SMA syndrome. Management is largely conservative in the first instance: duodenal and gastric decompression for symptom relief, fluid resuscitation, and correction of electrolyte imbalances, with aims of optimising nutrition via either enteral jejunal feed, TPN, or a combination of both, as was demonstrated in this case [1, 3]. This is especially important when the suspected pathophysiology is reduction of visceral fat. A number of surgical options have been described, such as gastrojejunostomy, duodenojejunostomy, or Strong's operation. However, surgical management is typically considered second line, following a trial of failed conservative therapy [1]. A range of surgical options have been described for the management of symptomatic patients in some instances.

## 4. Conclusion

SMA syndrome is a rare cause of upper gastrointestinal obstruction, which can have a fulminant or an insidious clinical course. Even when radiologically extreme, symptoms may not clinically correlate with the severity of the anatomical distortion, thus making the condition difficult to diagnose. It may be associated with the Nutcracker phenomenon, with or without its own clinical manifestations. Clinicians should be aware of this diagnosis and consider it in cases of abdominal pain that are not in proportion to biochemical or clinical findings.
